# The impact of a prolonged ebola outbreak on measles elimination activities in Guinea, Liberia and Sierra Leone, 2014-2015

**DOI:** 10.11604/pamj.supp.2020.35.1.19059

**Published:** 2020-01-06

**Authors:** Balcha Girma Masresha, Richard Luce, Goitom Weldegebriel, Reggis Katsande, Alex Gasasira, Richard Mihigo

**Affiliations:** 1 WHO, Regional Office for Africa, Brazzaville, Congo; 2 WHO, Inter-country team for Western Africa, Ouagadougou, Burkina Faso; 3 WHO, Inter-country team for Eastern and Southern Africa, Harare, Zimbabwe; 4 WHO, Country office for Zimbabwe, Harare, Zimbabwe

**Keywords:** Ebola, measles, vaccination, West Africa, coverage, outbreak, Liberia, Guinea, Sierra Leone

## Abstract

**Introduction:**

Guinea, Sierra Leone and Liberia have attained significant reduction in measles incidence between 2004 and 2013. The Ebola outbreak in 2014-2015 in West Africa caused significant disruption of the health service delivery in the three worst affected countries. The magnitude of the impact on the immunization program has not been well documented.

**Methods:**

We reviewed national routine immunization administrative coverage data as well as measles surveillance performance and measles epidemiology in the years before, during and after the EVD outbreak in Guinea, Liberia, Sierra Leone.

**Results:**

Both Liberia and Guinea experienced a sharp decline of more than 25% in the monthly number of children vaccinated against measles in 2014 and 2015 as compared to the previous years, while there was no reported decline in Sierra Leone. Guinea and Liberia experienced a decline in measles surveillance activity and performance indicators in 2014 and 2015. During this period, there was an increase in measles incidence and a decline in the mean age of measles cases reported in Liberia and Sierra Leone. Guinea started reporting high measles incidence in 2016. All three countries organized measles supplemental immunization activities by June 2015. Liberia achieved 99% administrative coverage, while Guinea and Sierra Leone attained 90.6% and 97.2% coverage respectively. There were no severe adverse events reported during these mass vaccination activities. The disruptive effect of the Ebola outbreak on immunization services was especially evident in Guinea and Liberia. Our review of the reported administrative vaccination coverage at national level does not show significant decline in measles first dose vaccination coverage in Sierra Leone as compared to other reports. This may be due to inaccuracies in coverage monitoring and data quality problems. The increases in measles transmission and incidence in these three countries can be explained by the rapid accumulation of susceptible children. Despite the organization of mass vaccination activities, measles incidence through 2017 has remained higher than the pre-Ebola period in all three countries.

**Conclusion:**

The Ebola outbreak in West Africa significantly affected measles vaccination coverage rates in two of the three worst affected countries, and led to persistent gaps in coverage, along with high measles incidence that was documented until two years after the end of the Ebola outbreak. Liberia and Sierra Leone have demonstrated coverage improvements after the end of the Ebola outbreak.

## Introduction

In 2011, Member States of the WHO African Region established a goal to achieve measles elimination by 2020 [1]. The strategies to achieve elimination include increasing access and measles vaccination coverage with routine immunization services in all districts; achieving high coverage during all measles Supplemental Immunisation Activities (SIAs), as well as outbreak response immunization activities, improving the quality of measles surveillance and rapidly investigating measles outbreaks in all countries. The Member States adopted a goal comprised of the following targets: (i) ≥ 95% coverage with the first dose of measles-containing vaccine (MCV1) at national and district levels; (ii) ≥ 95% coverage in all districts during measles SIAs; and (iii) confirmed measles incidence < 1 per million population in all countries. (iv) Conducting high quality measles surveillance defined as ≥ 2 cases of non-measles febrile rash illness (NMFRI) per 100,000 population annually and collecting a blood specimen from ≥ 1 suspected measles case in ≥ 80% of districts annually [1]. The measles elimination goal is also an objective of the African Regional Immunization Strategic Plan 2014 – 2020 [2]. By the end of 2017, the African Region of the WHO attained 86% reduction in the estimated mortality from measles as compared to estimated measles mortality for 2000 [3].

Guinea and Sierra Leone began implementing measles control strategies in 2003 when both countries implemented their initial national measles supplemental immunization activities (SIAs) targeting children aged 9 months to 14 years of age, while Liberia had its initial measles SIAs in 2004. All three countries established case-based surveillance for measles supported by serological testing of suspected cases by the end of 2004. Since then, these three countries have made considerable progress controlling measles. They reported officially to the WHO a total of 96,910 measles cases in the 10 years period from 1994 to 2003, while this number declined sharply to a total of 6,937 over the 10 years period between 2004 and 2013 [4]. The Ebola virus disease (EVD) outbreak in 2014-2015 in West Africa was the largest Ebola epidemic ever documented. Between December 2013 and April 10, 2016, a total of 28,616 suspected, probable, and confirmed cases of Ebola virus and 11,310 deaths were reported, of which all but 36 cases were from the three countries. The peak period of Ebola case reporting was in the second half of 2014 in Liberia, while Sierra Leone continued to report many cases in the first quarter of 2015, and transmission continued until the third quarter of 2015 in Guinea. The Ebola outbreaks in Liberia, Sierra Leone, and Guinea ended in May, November, and December 2015 respectively [5, 6]. During the period of intense Ebola transmission in the three countries, many health facilities were closed, and others operated at lower capacity than usual, because of shortage of staff and disruption of medical logistics supplies. In addition, health service utilization declined significantly due to fear of acquiring Ebola infection at health facility settings, the shifting of health resources towards the Ebola response, and due to the death of health care staff [7].

Routine immunization services, previously scheduled SIAs and the introductions of new vaccines, as well as supervisory visits and program reviews were cancelled or postponed as health systems were overwhelmed by the scale of the Ebola outbreak and the magnitude and duration of response efforts [7, 8]. Studies have also documented the decline in maternal and child health services in Guinea, as well as curative services in Sierra Leone [9–11] Others have modelled the expected increase in deaths from diseases such as malaria, as a result of significant reduction in the availability of treatment services in health facilities [12]. Measles has been previously recognized as an important communicable disease to anticipate during disasters and humanitarian crises that result in population displacements and in the disruption of health systems [13]. Takahashi et al have modelled the increased susceptibility to measles resulting from the Ebola epidemic in West Africa [14], while others have highlighted the programmatic difficulties in maintaining routine vaccination services [15]. Measles outbreaks have been documented in the three countries during and after the Ebola epidemic [16, 17]. Suk et al reported on 284 cases of measles from January 23, 2015–April 4, 2015 in Lola prefecture in Guinea, with the average and median age of patients being 2.8 years and 2.0 years of age, and with 95% cases not having been vaccinated [17].

With the prolonged disruption of immunization and health services, the risk for outbreaks of vaccine preventable diseases was recognized and WHO issued specific guidance to immunization programs in the region affected by Ebola in March 2015 [18]. The recommendation proposed that intensified routine vaccination activities and/or vaccination campaigns should be conducted, subject to certain conditions, when a risk assessment indicates that risk of vaccine-preventable disease outbreaks (i.e. measles, etc.) outweighs the risk of increased Ebola virus transmission. This manuscript examines the immunization program and surveillance data from Guinea, Liberia and Sierra Leone, and quantifies the impact of the EVD outbreak on service delivery, surveillance performance and measles disease burden in the three countries.

## Methods

We conducted a review of secondary data available with the WHO Regional office for Africa. The datasets we reviewed included national routine immunization administrative coverage data as well as measles surveillance performance and measles epidemiology data in the years before, during and after the EVD outbreak in Guinea, Liberia, Sierra Leone. These datasets are shared with the WHO by Member States regularly, for purposes of monitoring of trends and performance, as well as for assistance with analysis and feedback. Analysis of data was done using MS Excel and Epi Info software.

**Routine immunization coverage:** in these countries, vaccination coverage is determined by recording the number of children who receive each vaccine antigen on paper reporting forms in every service delivery point in the health system. Data on children vaccinated is aggregated and entered into a database at the district level for onward transmission and compilation at the national level. The national level shares the compiled country data with the WHO as a monthly report detailing the monthly number of children vaccinated by antigen and by district. We reviewed the routine immunization coverage administrative data to analyze the monthly number of children who received measles vaccine for the years 2012 – 2017. WHO and the United Nations Children’s Fund (UNICEF) estimate vaccine coverage for each country and each antigen by conducting a country-by-country review of administrative data, data from surveys and other sources. These estimates are published annually on the WHO website, and are updated as additional data becomes available [19]. We reviewed the WHO UNICEF national coverage estimates for DPT3, yellow fever and the first dose of measles vaccine for the three countries over the years 2012 – 2017.

**Coverage in Supplemental Immunization Activities:** the Measles and Rubella Initiative and the Global Alliance for Vaccines and Immunization support countries to conduct periodic measles SIAs to increase population immunity against measles. At the end of the SIAs, countries submit technical reports to the WHO, detailing administrative coverage results and lessons learned. In most cases, post-campaign coverage surveys are implemented immediately after the end of the SIAs and survey reports are shared. We reviewed national SIAs technical reports and post-campaign coverage survey reports available with the WHO Regional Office for Africa to assess coverage levels [20].

**Case based surveillance performance and epidemiological trends:** we examined data from the case-based measles surveillance system in all three countries for the period 2012 - 2017. The measles case definition used to report suspected cases in the case-based surveillance system is: fever and generalized maculopapular rash plus one of the following clinical symptoms: cough, runny nose, or red eyes. For each suspected measles case, an investigation form was completed, a blood specimen was collected and sent to the national laboratory for measles specific immunoglobulin M (IgM) antibody testing. Suspected measles cases were confirmed by laboratory when there is serological confirmation of recent measles virus infection (measles IgM positive). In the case of lab confirmed measles outbreaks, cases may also be confirmed by epidemiological linkage. A clinically compatible case of measles is a suspected measles case that does not have a blood specimen taken for serologic confirmation and is not linked to any measles outbreak [21]. Surveillance performance was monitored using standard performance indicators. The two principal performance indicators are the non-measles febrile rash illness rate (target of at least 2 per 100 000 population) and the proportion of districts that have investigated at least one suspected case of measles with blood specimen per year (target at least 80% of districts per year). The incidence of confirmed measles was calculated as a rate per million, by dividing the total number of confirmed measles cases (confirmed by laboratory, epidemiological linkage and clinical criteria) by the total population [21].

## Results

### MCV1 coverage Liberia

According to the administrative coverage data, an average of 9332 children get vaccinated with MCV1 for the period January 2012 – Dec 2017 in Liberia. However, between August and November 2014, the number of children vaccinated with MCV1 declined, ranging between 3196 and 4494, which is more than 2 standard deviations from the period average of 9332 (SD = 2371). Compared to the monthly mean for 2012 (prior to the ebola outbreak), the mean monthly number of children vaccinated with MCV1 declined by 30% in 2014 and by a further 25% during 2015. By the end of 2017, the monthly mean number of children vaccinated with the first dose of measles vaccine showed a 13% increase compared to 2012 (Table 1). This decline was also reflected in the data from the WHO UNICEF estimates for national coverage with MCV1 in Liberia, where the 2-year mean MCV1 coverage in 2014 and 2015 (corresponding to the Ebola outbreak period) was 16% lower than the mean for the previous 2-year period (2012 – 2013). By the post-Ebola period of 2016-2017 Liberia’s MCV1 coverage was 84% as compared to the average of 77% for the years 2012-13 corresponding to the two year pre-ebola period (Table 2)

**Table 1:** Monthly mean of children vaccinated with MCV1 by year, and relative change compared to the mean for 2012

Monthly mean number of children receiving MCV-1 nationally						
Year	Guinea		Liberia		Sierra Leone	
	Mean	Change against 2012 levels	Mean	Change against 2012 levels	Mean	Change against 2012 levels
2012	34882	―	10084	―	16998	―
2013	34231	−2%	9483	−6%	18657	10%
2014	23403	−33%	7100	−30%	17139	1%
2015	25669	−26%	7583	−25%	17459	3%
2016	31885	−9%	10390	3%	20772	22%
2017	34394	−1%	11353	13%	19369	14%

**Table 2:** WHO UNICEF estimates of annual coverage for DPT3, yellow fever vaccine and MCV1, by country

		DPT3	YFV	MCV1	2 year mean MCV1 coverage
Guinea	2012	53%	51%	51%	45%
	2013	44%	39%	39%	
	2014	34%	28%	28%	38%
	2015	45%	43%	48%	
	2016	45%	43%	48%	48%
	2017	45%	43%	48%	
Liberia	2012	80%	78%	80%	77%
	2013	76%	73%	74%	
	2014	50%	54%	58%	61%
	2015	52%	56%	64%	
	2016	79%	73%	80%	84%
	2017	86%	84%	87%	
Sierra Leone	2012	91%	80%	86%	86%
	2013	92%	80%	85%	
	2014	83%	78%	80%	79%
	2015	86%	80%	78%	
	2016	84%	85%	85%	83%
	2017	90%	85%	80%	

### Guinea

In Guinea, the administrative coverage data shows that the mean monthly number of children who received MCV1 in the period January 2012 – Dec 2017 is 30744 (Standard deviation = 9557). This monthly average declined by 33% in 2014 and by 26% in Guinea in 2015 and remained 1% below the 2012 level by the end of 2017. In the last four months of 2014 and in December 2015, the number of vaccinated children ranged between 1195 and 4513, which is 2 standard deviations below the monthly mean of 30744 (SD = 9557) for the entire period. The WHO UNICEF national coverage estimates also show that the 2-year mean MCV1 coverage in 2014 and 2015 (corresponding to the Ebola outbreak period) was 7% lower than the mean for the previous 2-year period (2012 – 2013) in Guinea. After the end of the Ebola outbreak, the MCV1 coverage estimates in Guinea was higher (48% average for 2016-2017) as compared to 2012 - 2013 (45% coverage). On the other hand, the coverage estimates for DPT3 and YF vaccination coverage showed a decline (Table 2).

### Sierra Leone

**Figure 1:**
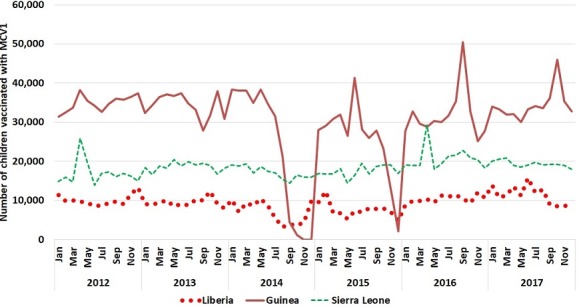
Monthly number of children vaccinated with MCV1 by country - January 2012 - December 2017

The national immunization program in Sierra Leone reached an average of 18399 (Standard deviation = 2431) children with MCV1 monthly between January 2012 and December 2017, according to the routine immunization administrative coverage data. This data shows that none of the monthly records of vaccinated children showed a decline below 2 standard deviations from the monthly mean at any time (Figure 1). On the other hand, compared to the reported data in 2012, the mean monthly number vaccinated in 2014 - 2015 increased slightly by 1-3%, and had a 14% increase by the end of 2017 as compared to 2012 (Table 1). The MCV1 coverage estimate in Sierra Leone showed a 3% decline from an average of 86% for 2012 – 2013 to 83% in 2016 – 2017 (Table 2).

### Supplemental Immunization Activities (SIAs) in the Ebola period

#### Liberia

Confronted with the deleterious effect of the Ebola outbreak on the health system, Liberia postponed a measles SIA scheduled for November 2014. In January 2015, after more than 6 months of disruption in health care delivery service including routine vaccination and a corresponding decline in the number of children receiving vaccination, the risk for measles outbreaks increased with reports of measles outbreaks in some areas. As a result, Liberia organized Periodic Intensification of Routine Immunization (PIRI) activities to selectively reach unvaccinated children aged 9 – 59 months vaccinating 109,069 children across 13 of the 15 counties. In 2 of the 13 counties, the intervention was limited to providing vaccination only to unvaccinated infants 9 – 11 months of age. The postponed nation-wide measles SIA was conducted in May 2015 and was integrated with the administration of oral polio vaccination, deworming and Vitamin A supplementation). The campaign targeted 596,545 children aged 6 - 59 months of age and achieved 99% administrative coverage. The post-campaign coverage survey showed that national coverage was 90.4%, while subnational coverage ranged from 99.2% in Grand Gedeh County to 72.4% in Grand Bassa County.

**Guinea:** in the face of intensifying measles transmission and outbreaks, Guinea organized outbreak response vaccination campaigns in 2 phases between February and April 2015, and vaccinated 1,259,690 children 6 months to 10 years of age in 263 centres de sante across 15 of 21 provinces attaining administrative coverage of 90.6% at national level. This activity was integrated with Vitamin A supplementation and Mebendazole administration to children under 5 years of age. Another measles follow-up SIAs was organized in February 2016 and reached 2,412,923 children 9 – 59 months of age in 38 districts across 8 provinces, attaining 102.7% administrative coverage. Post-campaign coverage survey results indicated coverage of 92.7% (95% CI: 92.1% - 93.2%).

**Sierra Leone:** Sierra Leone organized a nation-wide measles SIA in June 2015 vaccinating 1,205,865 children from 9 – 59 months of age and achieving 97.2% administrative coverage. The SIA was integrated with OPV administration for children less than 5 years of age, as well as the identification of children who had missed certain vaccines and provision of other antigens to eligible infants. Following the occurrence of continued outbreaks involving children over 5 years of age, Sierra Leone conducted a wide age range nationwide measles immunization in May 2016 which reached 2,795,686 children aged 6 months to 14 years and achieving 100% administrative coverage. The post-campaign coverage survey results was 97.7% at national level (95% CI: 97.2% - 98%). The survey reported that 20.2% of the children vaccinated in the campaign received measles vaccination for the first time.

### Case based measles surveillance

#### Liberia

In Liberia, the proportion of districts reporting suspected cases with blood specimens decreased from 88% in 2012 to 44% in 2013 and to 6% in 2014. The non-measles febrile rash illness rate (NMFRI rate) for 2014 and 2015 also declined by 20% in Liberia as compared to the 2-year averages for 2012 and 2013. Liberia missed the NMFRI target of 2 per 100,000 in all 6 years from 2012-2017. Liberia achieved the district reporting target only in 2017 (Table 3). In Liberia, there were no confirmed measles cases reported through the case based surveillance system in 2013-14, but measles incidence rose to 108.5 per million in 2015. The mean (5.3 years) and median ages (3 years) of cases was lowest in 2015 as compared to the other years.

**Table 3:** Measles surveillance performance, measles incidence and age patterns by country, 2012 - 2017

	Year	% districts reporting (target ≥ 80%)	NMFRI rate per 100,000 population (target ≥ 2:100,000)	# of suspected measles cases	Blood specimens collected	# confirmed measles	Measles incidence/ million population	% confirmed measles aged < 5 years	Mean (median) age of confirmed measles cases
Guinea	2012	95%	1.0	140	140	7	0.6	43%	4.5 (5) years
	2013	97%	0.9	163	163	39	3.3	74%	3.5 (2) years
	2014	97%	0.4	266	266	35	2.9	83%	3.1 (2) years
	2015	39%	0.2	48	48	29	2.7	61%	4.2 (3) years
	2016	97%	2.7	636	628	128	11.5	65%	4 (3) years
	2017	100%	6.1	1268	1268	583	52.5	70%	4.2 (3) years
Liberia	2012	88%	1.0	42	41	4	0.1	67%	12.6 (7) years
	2013	44%	0.5	20	20	0	0.0		
	2014	6%	0.1	3	3	0	0.0		
	2015	13%	1.1	479	12	436	108.5	60%	5.3 (3) years
	2016	31%	1.1	449	269	400	97.4	43%	9.4 (5) years
	2017	88%	0.4	409	328	96	23.4	27%	8.9 (7 years)
Sierra Leone	2012	93%	1.2	123	123	42	0.6	56%	5.8 (4) years
	2013	93%	0.6	59	59	13	2.1	62%	4.4 (4) years
	2014	93%	1.7	150	150	44	6.9	68%	4.2 (2.5) years
	2015	93%	1.9	266	266	128	18.0	61%	6 (4) years
	2016	87%	2.9	414	414	195	26.7	46%	7.4 (5) years
	2017	93%	6.2	536	536	76	10.4	22%	7.8 (7) years

#### Guinea

Guinea experienced a decline in the proportion of districts reporting suspected measles cases from 95% in 2012 to 39% in 2015. in addition, Guinea had a significant reduction in the non-measles febrile rash illness rate (NMFRI rate) for 2014 and 2015 (declined by 68% as compared to the 2-year averages for 2012 and 2013). In 2016 and 2017, Guinea attained the targets for both principal performance indicators (Table 3). In 2014, 84% of the confirmed measles cases were less than 5 years of age while the mean age of confirmed cases (3.1 years) was the lowest during the period. In Guinea, the incidence of measles increased from 2.7 per million in 2015 to 11.5 per million in 2016.

#### Sierra Leone

**Figure 2:**
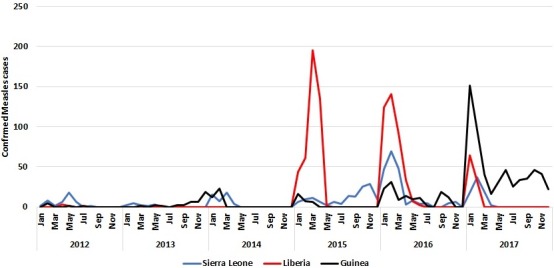
Monthly trends of confirmed measles cases by country January 2012 - December 2017

The proportion of districts collecting blood specimens from suspected measles cases did not show a decline in Sierra Leone in the years of the Ebola outbreak. Sierra Leone did not meet the target of 2 per 100,000 NMFRI rate in 2012 – 2015. the country managed to attain the targets for both principal performance indicators in 2016 and 2017. The incidence of measles increased from 6.9 per million in 2014 to 18 per million in 2015 in Sierra Leone. The proportion of confirmed measles cases less than 5 years of age was greatest in 2014 (68%) as compared to the other years, and mean (4.2 years) and median ages (2.5 years) were the lowest during the period. Confirmed measles incidence increased markedly in all three countries around the time of the Ebola outbreak and remained high in 2016 and 2017. The monthly trend of reported measles indicates that all three countries had increased case reporting in the first 4 months of the calendar year (Figure 2).

## Discussion

Guinea, Liberia and Sierra Leone experienced protracted civil conflict in the last 20 years. These countries have low developmental indices and very low scores for most of the health system matrices [22]. These factors contributed to the unprecedented scale and duration of the EVD outbreak in west Africa. The resulting disruption of routine health care delivery systems was severe and had multiple social-behavioral, logistical, and economic dimensions [7, 8]. Nationwide health emergencies put already fragile health systems under stress. As health services are disrupted, vaccination services fail to reach children resulting in an accumulation of infants and young children who are not protected against measles, diphtheria and other vaccine preventable diseases. At the same time, the health system’s capacity to detect, notify and confirm reported cases declines sharply complicating efforts to do meaningful and complete epidemiological analysis of the situation [8, 15, 23]. Because of the highly infectious nature of measles, large and explosive measles outbreaks often occur early in the course of conflicts, natural disasters or other political crises that cause a disruption of health systems [13, 24].

The disruptive effect of the Ebola outbreak on immunization services was especially evident in Guinea and Liberia. From the administrative coverage data during the peak of the EVD transmission, Guinea experienced an extreme decline in the number of children vaccinated by routine services with MCV1, especially in the second half of 2014. Administrative coverage followed a similar dynamic in Liberia, while Sierra Leone experienced a smaller reduction in the number of children vaccinated in the 2014-2015 period. This is also reflected in the WHO- UNICEF coverage estimates, where Sierra Leone had smaller Ebola related decreases in coverage; however MCV1 coverage levels had not returned to pre-Ebola levels in 2017 [19]. Nonetheless, Elston et al. have reported a more than 50% decline in the monthly mean number of children receiving all recommended childhood vaccinations in the second half of 2014 as compared to January–June 2014 in Koinadugu district in Sierra Leone [7]. Similar declines were reported by the government of Sierra Leone in the proceedings of the Regional workshop on building resilient health systems in April 2016 [8]. Our review of the reported administrative vaccination coverage at national level does not show significant decline in measles first dose vaccination coverage in Sierra Leone. This may be due to differences in coverage changes across districts, or as a result of relatively high coverage maintained with one dose measles vaccination as compared to other antigens, or to the gaps in the completion of the primary series of antigens as reported by Elston et al. This discrepancy could be attributable to inaccuracies in administrative immunization coverage reporting. The combined Ebola-related drop in routine immunization coverage against existing sub-optimal coverage reflected in the WHO-UNICEF estimates, suggests that increases in measles transmission and incidence were probable in these three countries due to insufficient population immunity and a rapid accumulation of susceptible children. An increase in the incidence of confirmed measles was identified in each of the three countries starting in the 2015-2016 period. Despite the organization of mass vaccination campaign and outbreak response vaccination activities, confirmed measles incidence through 2017 has remained higher than the pre-Ebola period in all three countries. These results emphasize the lasting effects of persistent weakness in the provision of immunization service from the time of the Ebola outbreak and improved performance of surveillance in the post-Ebola period.

In addition, the age of measles cases was comparatively lower in the period 2014 – 2015, also suggesting a disruption in vaccination services that may have left young children unvaccinated which is likely a result of a rapid accumulation of unvaccinated susceptible children occurring at the peak of the EVD outbreak and in the months immediately after the end of the outbreak, when efforts to rebuild the health systems were still in the early stages. This indicates the prolonged impact of acute and severe health system failure that resulted in lingering insufficiency in population immunity to measles and other VPDs. The disruption of essential health services during the EVD outbreak was also documented in the area of maternal and child health services in Guinea, with declines in the number of institutional deliveries and frequency of antenatal visits and in the declines in the number of hospital admissions and surgical procedures in Sierra Leone [9–11]. The Ebola-related decline in measles surveillance performance was also more pronounced in Guinea and Liberia. The decline in surveillance quality, decline in health-care seeking, as well as the inability to collect and ship specimens for testing combined to underestimate actual incidence levels. As measles surveillance was re-established, case detection and confirmation improved at the same time that measles transmission intensified. The guidance from WHO on immunization during the Ebola outbreak recommended intensified routine vaccination activities and/or vaccination campaigns if programmatic assessment shows a risk of vaccine-preventable disease outbreaks [18]. The guidance specifically suggested that countries with intense and widespread transmission of Ebola virus implement crowd control, triage, infection prevention and control measures when conducting vaccination activities, as well as observing safe injection and waste disposal practices.

All three countries organized measles campaigns within the Ebola period due to outbreaks and surveillance data that confirmed measles transmission and heightened risks. Vaccine hesitancy was reported in various districts of all three countries due to the fear of acquiring Ebola infection via injection. Intensified community engagement and dialogue with traditional and religious leaders was employed to gain acceptance of the campaigns. Liberia and Guinea reported challenges in conducting the campaigns due to insufficient number of health staff to act as supervisors. Special provisions were made in all 3 countries to assure injection safety during the campaigns. Only trained and qualified health workers were engaged to administer the vaccine. Vaccination teams were supplied with auto-disable syringes for injection, safety boxes for the disposal of sharps, as well as hand sanitizers, gloves and aprons to observe recommended infection prevention and control procedures. There were no reports of ebola contamination or transmission resulting from injection practices during the supplemental immunization activities and no severe cases of adverse event following immunization were reported. The measles SIAs in early 2015 in all three countries were the first large-scale immunization interventions conducted during an ongoing Ebola outbreak. The experience of organizing measles SIAs during an Ebola outbreak and achieving high coverage, indicates that mass vaccination campaigns can be effectively undertaken in such conditions with appropriate planning and precautions to assure safe injection practices to prevent Ebola transmission.

In addition, when rebuilding damaged health systems, immunization remains a cost-effective first-line priority intervention and should be re-established with a view to provide timely and complete protection to the most vulnerable segments of the population against vaccine preventable diseases [25]. The coverage improvements documented in Liberia and Sierra Leone in 2016 – 2017 demonstrate that focusing on immunization in the agenda to rebuild health systems can be effective. This analysis is subject to limitations. First, the completeness and reporting of administrative immunization coverage data was negatively affected by the ebola outbreak and may report a greater drop in numbers than actually occurred in health facilities. Second, where surveillance performance decreased, the ability of the health system to detect and confirm suspected cases was adversely impacted potentially resulting in under reporting of the measles cases and actual measles incidence.

## Conclusion

The immunization service delivery was affected early in the course of the Ebola outbreak in the three worst affected countries in West Africa, and led to persistent gaps measles immunization coverage and high measles incidence that was documented until two years after the end of the Ebola outbreak. All three countries implemented measles outbreak response and supplemental immunization activities with the necessary precautions. The reporting and investigation of measles cases improved in the immediate post-Ebola period, while Liberia and Sierra Leone have demonstrated coverage improvements after the end of the Ebola outbreak, attesting to the high level programmatic attention paid to immunization in the health system rebuilding efforts.

### What is known about this topic

- Guinea, Liberia and Sierra Leone had weak health systems before the EVD outbreak, which was further impacted negatively with the EVD outbreak;
- The EVD outbreak in 2014 – 2015 significantly disrupted health services in the country’s worst affected, including childhood immunization services;
- Periodic supplemental immunisation activities (SIAs) are essential in order to close immunity gaps created through suboptimal routine immunisation coverage and as a result of disruption of health service delivery.

### What this study adds

- This study quantifies the degree of disruption of the immunization services during and after the EVD outbreak in West Africa;
- Disease surveillance systems were disrupted at the same time as immunisation service delivery, and were not able to provide sensitive and timely indication of the immunity gaps and the increasing transmission of measles in the EVD affected countries;
- The measles SIAs were the first major immunisation interventions implemented in the three countries affected by the EVD outbreak, and were conducted with appropriate caution to avert the occurrence of AEFIs, and respecting the infection prevention and control measures in place to limit the spread of EVD.

## Competing interests

The authors declare no competing interests.
